# 

**Published:** 1874-05

**Authors:** 


*American Medical Association.*

The twenty-fifth annual session of this body will be held in the city of Detroit, Michigan, on Tuesday, June 2d, 1874. Our views are well known in regard to the objects of this Association. We have been its warmest and strongest defenders at all times and under all circumstances. We are willing to throw the veil of charity over its errors and defects, and bid it God-speed in its noble objects. We deeply regret that circumstances preclude the possibility of our being present at the ensuing meeting. We trust it will be the largest meeting we have ever had, and that many abuses and errors may be corrected; that many steps may be taken forward and upward in the march of progress. The first step towards the correction of abuses is the intimation of their existence. If the members and friends of the Association fail to attend—and occasionally men of mediocre minds and worse morals get admittance and control, misrepresenting the profession of their respective States—the fault is not to be attributed to the body as an Association. Indifference and a little tricking has ruined many a good cause, and we (with scores of the best men in every section) fear, but we will not say more. "All is well that ends well," and we hope the best even when hope promises the least. P.

NOTE.—Book notices and receipts crowded out in this issue.

### ASSOCIATE EDITORS:

GEORGIA.

James A. Long, M.D.
W. L. M. Harris, M.D.
H. L Battle, M.D.
W. C. Musgrove, M D
L. B. Bouchelle, M.D.
Thomas Burdell, M.D.
E. H. W. Hunter, M.D.
V. S Hitt, M.D.
J. E. Pope, M.D.
A. H. Brantley. M.D.
J. J. Knott, M.D.
J. C. Wisdom, M.D.
W. W. Leake, M.D.
S. G. WHITE, M.D.
W. H. HALL, M.D.
G. D. CASE, M.D.

KENTUCKY.

W. S. Taylor, M.D.
B. W. Stone, M.D.
Charles Kearns, M.D.

TEXAS.

S. M. Welch, M.D.
T. J. Heard, M.D.
W. A. Heard, M.D.

TENNESSEE.

J. D. Tucker, M.D.

ALABAMA.

J. C. Knox, M.D.
Geo. F. Taylor, M.D.
J. B. Barnett, M.D.
S. M. Hogan, M.D.
D. S. Hopping, M.D.
J. T. Searcy, M.D.
S. F. Hill, M.D.

NORTH CAROLINA.

J. B. Hughes, M.D.
S. S. Satchwell, M.D.
A. B. Pierce, M.D.
H. Kelly, M.D.
Geo. A. Foote, M.D.
E. A. Anderson, M.D.
H. T. Bahnson, M.D.
Willis Alston, M.D.
Thos. F. Wood, M.D.
N. J. Pittman, M.D.
John W. Booth, M.D.

SOUTH CAROLINA.

E. T. McSwain, M.D.
G. M. Rivers, M.D.

OHIO.

J. Q. A. Hudson, M.D.
C. H. Tidd, M.D.

VIRGINIA.

John H. Claiborne, M.D.
F. Horner, Jr., M.D.
R. J. Preston, M.D.
J. E. Lockridge, M.D.
J. S. Wharton, M.D.
Wm. O. Owen, M.D.
Sam'l P. Ripley, M.D.
Benj. Blackford, M.D.
E. A. Drewry, M.D.
Rob B. Tunstall, M.D.
W. F. Barr, M.D.
L. B. Anderson, M.D.
J. M. Lazzell, M.D.

MISSISSIPPI.

C. B. Gallaway, M.D.
Edward Lea, M.D.
S. V. D. Hill, M.D.
E. T. Henry, M.D.
T. P. Lockwood, M.D.
Robert Kells, M.D.

ARKANSAS.

A. D. Hodge, M.D.

J. K. Hodge, M.D.

A. W. Hobson, M.D.

NEW JERSEY.

J. B. MATTISON, M.D.

### CORRESPONDING EDITORS:

Ely Van DeWarker, M. D., New York.
C. C. F. Gay, M.D., New York,
*Surgeon of Buffalo General Hospital.*
M. H. Henry, M.D., New York,
*Surgeon to N. Y. Dispensary, Dep't of Venerial and Skin Diseases.*
Benjamin Howard, M.D., New York.
James Tyson, M.D., Philadelphia, Pa.
W. W. Keen, M.D. Philadelphia.
Geo. Strawbridge, M.D., Philadelphia.
Robert Robson, M.D., Indiana.
A. Patton, M.D., Indiana.
John H. Weir, M.D., Illinois.
Thomas J Gallaher, M. D, Pennsylvania.
S. C. Busey, M.D., Washington, D. C.
Wm. R. Whitehead, M.D., Colorado Territory.

☞ The RECORD will be mailed between the 20th and 25th of each month.

